# Correction: Bräuchle et al. Large-Scale Expansion of Suspension Cells in an Automated Hollow-Fiber Perfusion Bioreactor. *Bioengineering* 2025, *12*, 644

**DOI:** 10.3390/bioengineering12101109

**Published:** 2025-10-15

**Authors:** Eric Bräuchle, Maria Knaub, Laura Weigand, Elisabeth Ehrend, Patricia Manns, Antje Kremer, Hugo Fabre, Halvard Bonig

**Affiliations:** 1 Institute for Transfusion Medicine and Immunohematology, German Red Cross Blood Service Baden-Württemberg-Hessen, 60528 Frankfurt, Germany; l.weigand@blutspende.de (L.W.); e.ehrend@blutspende.de (E.E.); p.manns@blutspende.de (P.M.); h.boenig@blutspende.de (H.B.); 2 Faculty of Biological Science, Goethe University, 60439 Frankfurt, Germany; 3 Terumo Blood and Cell Technologies, 1930 Zaventem, Belgium; maria.knaub@terumobct.com (M.K.); antje.kremer@terumobct.com (A.K.); hugo.fabre@terumobct.com (H.F.); 4 Faculty of Medicine, Goethe University, 60528 Frankfurt, Germany; 5 Department of Medicine, Division of Hematology and Oncology, University of Washington, Seattle, WA 98103, USA

## Missing Funding

In the original publication [1], the funding statement read as follows: “**Funding:** This research received no external funding.”

The authors state that the funding information was erroneously omitted. The following statement is correct: “**Funding:** The studies were supported by research grants 2021_EKTP08 and 2022_EKTP10 from ForTra gGmbH for Research Transfer of the Else Kröner-Fresenius Foundation to HB.”

The authors state that the scientific conclusions are unaffected. This correction was approved by the Academic Editor. The original publication has also been updated.
