# Supplementary material for: Large-Scale Expansion of Suspension Cells in an Automated Hollow-Fiber Perfusion Bioreactor
Source: Bioengineering (Basel). 2025 Jun 12;12(6):644. doi: 10.3390/bioengineering12060644 (PMC12189701; doi:10.3390/bioengineering12060644)
Supplement: Supplementary file 1 [file bioengineering-12-00644-s001.zip › bioengineering-3635501-supplementary.pdf]

bioengineering

Supplementary material

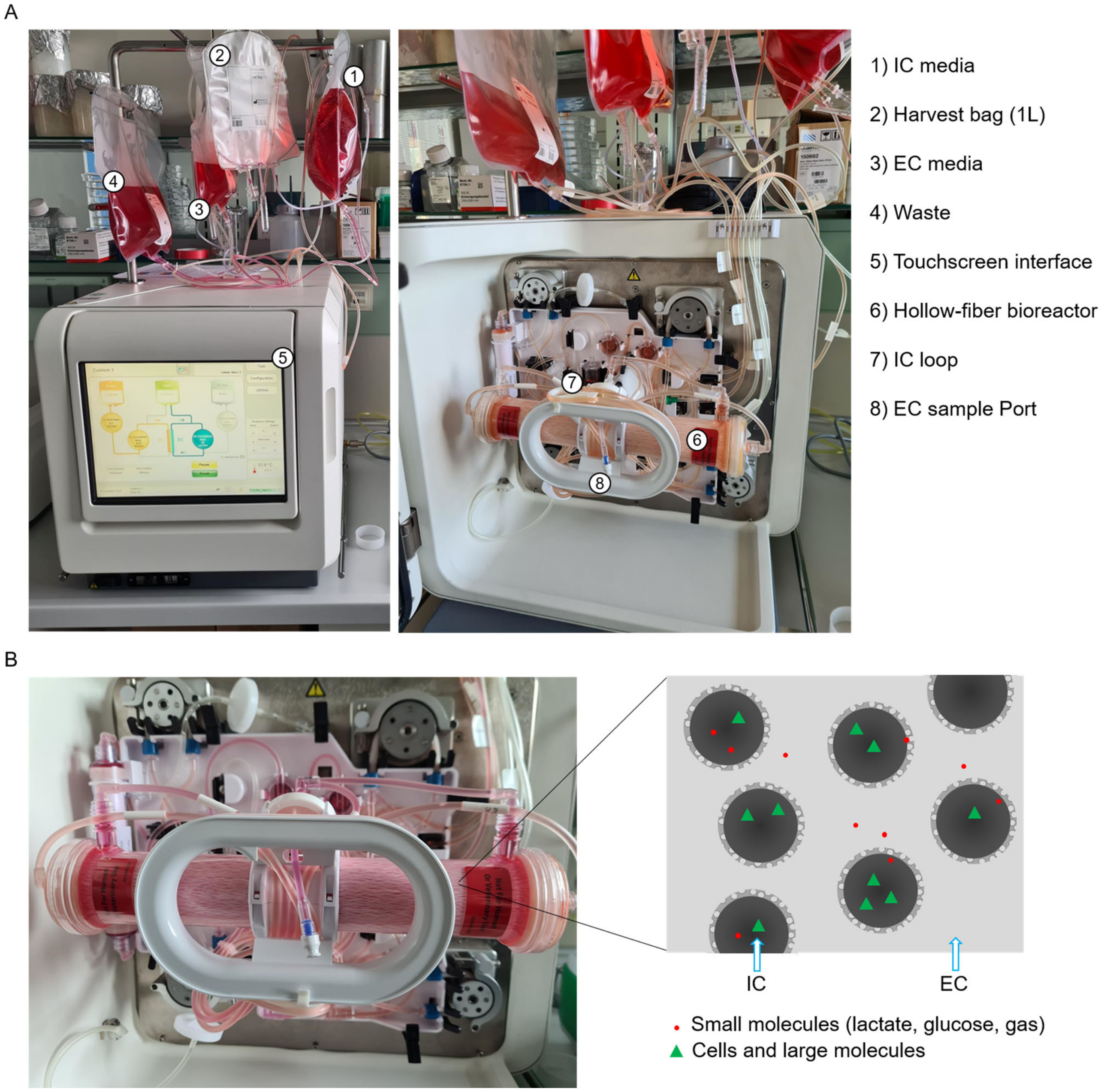

Figure S1. Automated expansion of suspension cells in the Quantum hollow-fiber perfusion bioreactor. (A) Quantum Cell Expansion System used for all automated expansion experiments in this study. The system with door closed (left) illustrates the setup with medium reservoirs for cell culture, waste, and harvest (1–4), as well as the touchscreen interface (5) for process control, including regulation of inlet flow rates and execution of harvest protocols. System with opened door (right) displays the integrated hollow-fiber bioreactor (6) within the system housing, along with peristaltic pumps and in-process control ports for extracapillary metabolite sampling (EC-sample port) (8) and intracapillary cell monitoring (IC-loop) (7). (B) Close-up of the hollow-fiber bioreactor unit during operation, showing the flow path and capillary arrangement. Schematic representation of the capillary system, highlighting the separation between the extracapillary (EC) and intracapillary (IC) compartments. Small molecules (red dots) can freely diffuse across the semipermeable membrane, while cells and larger molecules (green triangles) are retained within the IC space.
